# Zhoushi Qiling decoction induces apoptosis of human prostate cancer cells via miR-143/Bcl-2 axis

**DOI:** 10.18632/aging.203171

**Published:** 2021-06-25

**Authors:** Hongwen Cao, Dan Wang, Renjie Gao, Lei Chen, Yigeng Feng, Dongwen Gao

**Affiliations:** 1 Surgical Department I (Urology Department), Longhua Hospital Shanghai University of Traditional Chinese Medicine, Xuhui 200032, Shanghai, China; 2 Department of Ultrasound, Longhua Hospital Shanghai University of Traditional Chinese Medicine, Xuhui 200032, Shanghai, China

**Keywords:** traditional Chinese medicine, Zhoushi Qiling, prostate cancer, miR-143

## Abstract

A number of traditional Chinese medicines (TCMs) are widely used in prostate cancer treatment in China. The aim of this study was to test the efficacy of a TCM, Zhoushi Qiling Decoction (ZQD), in combination with androgen deprivation therapy (ADT) and explore its underlying mechanism. A total of 151 patients were recruited to receive ADT treatment or ADT+ZQD treatment. The survival of patients who received ADT+ZQD treatment was significantly higher than those who received ADT therapy only. DU145 prostate cancer cells were treated with ZQD (50 mg/mL) for 24 h *in vitro* and expression levels of an array of miRNAs were examined. Our results suggested that miR-143 demonstrated prominent upregulation in DU145 cells after treatment with ZQD. In patient serum samples, miR-143 expression was also significantly upregulated after ADT+ZQE treatment, which was however absent in patients treated with ADT only. In DU145 cells, ZQD treatment led to a dose-dependent increase in apoptosis, which could be reduced by anti-miR-143 treatment. There was a binding site between miR-143 and B cell CLL/lymphoma-2 (Bcl-2) and ZQD treatment reduced Bcl-2 expression. ZQD treatment led to increased caspase-3 and Bax expression. ZQD treatment could promote apoptosis of prostate cancer cells by promoting miR-143 upregulation, which could be a possible mechanism underlying the inhibitory effect of ZQD in prostate cancer in patient.

## INTRODUCTION

Prostate cancer is a highly prevalent cancer in the male urinary system [1]. For advanced prostate cancer, androgen-deprivation therapy (ADT) is a common treatment that could effectively delay disease progression at the beginning of the treatment [2]. However, the effectiveness of ADT varies among patients, and some develop hormone-independent prostate cancer (HIPC) at 14-30 months of treatment [3], rendering the treatment ineffective. The prognosis of patients with HIPC is abysmal (less than 20 months) and effective treatment is lacking at this disease stage.

To enhance ADT, a combinatory treatment strategy is often adopted. For example, chemotherapy drugs or androgen receptor inhibitors are used in combination with ADT to enhance the therapeutic efficiency [4]. However, the toxicity of these drugs is a significant obstacle for the application of these treatments. One of the combinational treatments commonly practiced in China is the addition of traditional Chinese medicine (TCM). Zhoushi Qiling decoction (ZQD), a TCM formula prepared by Prof. Zhou Zhiheng of Longhua Hospital, Shanghai University of Traditional Chinese Medicine, is one such treatments that showed promising efficacy in reducing the pain of endocrine therapy and prolonging survival of prostate cancer patients. ZQD was shown to prominently reduce bone metastasis of prostate cancer. ZQD treatment was also shown to improve prostate serum antigen (PSA) levels and Karnofsky Performance Status scores. But the clinical application of this TCM is hindered by the unclear mechanism of action, and how ZQD enhances ADT therapy in prostate cancer remains largely unknown.

Herein, the aim of our study was to elucidate the mechanism of ZQD in inhibiting prostate cancer. We focused our study on exploring microRNAs (miRNAs), which are short non-coding RNAs with a length of about 22 nucleotides, which post-transcriptionally modulate the expression of target genes [5]. Since a number of TCMs have been shown to attenuate cancer progression through regulating miRNAs, and many miRNAs are important genetic targets in prostate cancer, our study aimed to clarify miRNAs that are transcriptionally altered by ZQD therapy. We conducted a non-blinded open-labeled randomized clinical trial to confirm the effectiveness of ADT+ZQD therapy in prolonging prostate patient survival, using ADT treatment alone as a control. Through *in vitro* study and analysis of patient serum samples, we confirmed that miR-143 was significantly upregulated after ZQD treatment, which was concomitant with increased apoptosis of DU145 cells after ZQD treatment. Further, we investigated the potential targets of miR-143, and bioinformatics and luciferase assays suggested that an oncoprotein, B cell CLL/lymphoma-2 (Bcl-2), was a target of miR-143. Therefore, our results in the study could potentially advance the clinical application of ZQD by showing that ZQD could treat prostate cancer through the miR-143/Bcl-2 axis.

## RESULTS

### Study recruitment and patient baseline characteristics

**Figure 1:**
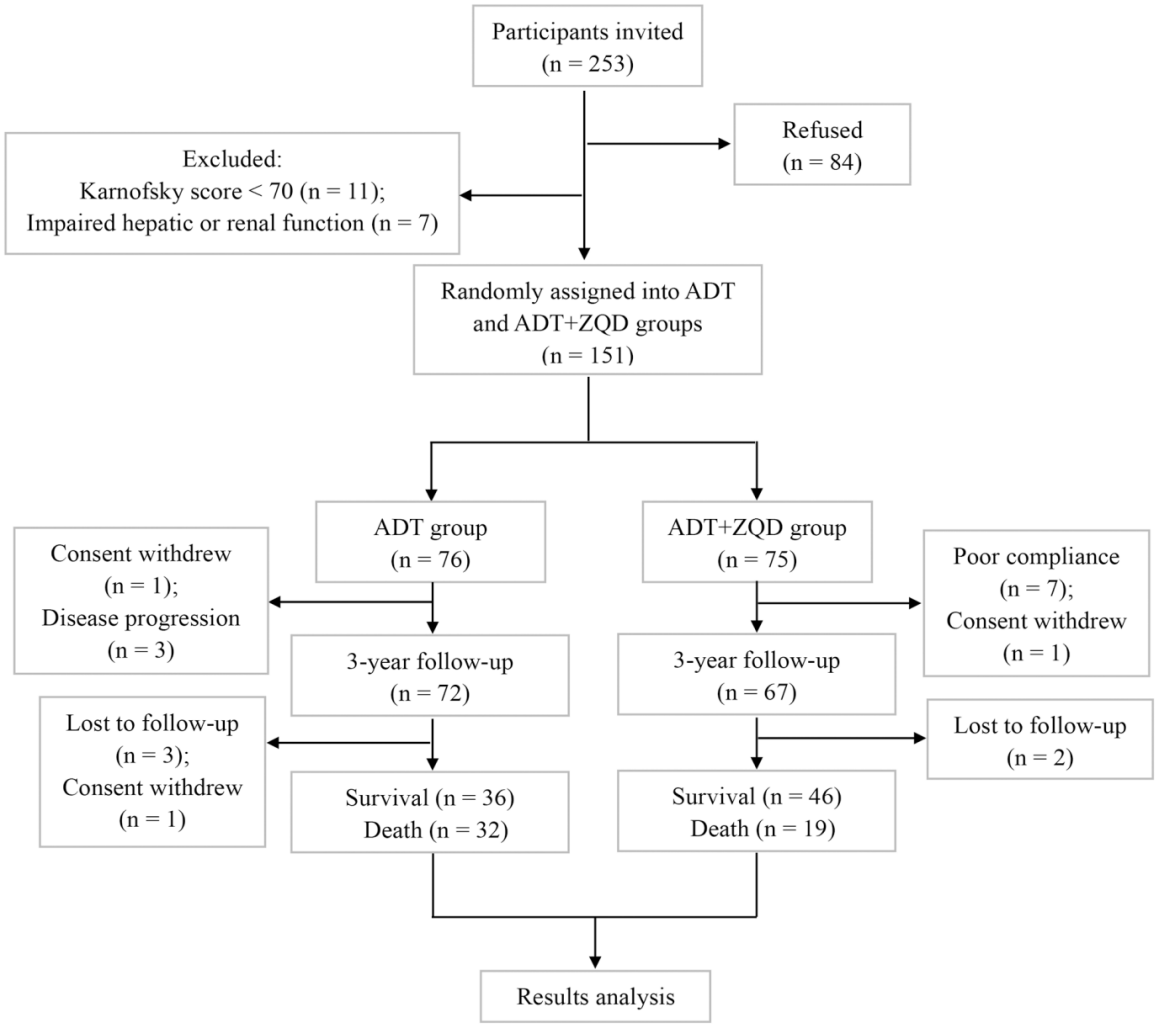
Flowchart of study participants.

The flowchart of study participants is shown in Figure 1. We recruited 253 patients, among which 11 were excluded due to low Karnofsky scores (<70) and 7 were excluded because of impaired hepatic or renal functions. Eighty-four patients refused to participate in the study. 151 patients were randomly assigned to ADT group (n=76) or ADT+ZQD group (n=75). The treatment lasted for three years and the survival rate of patients was assessed. Among the patients for analysis (n=68 for ADT and n=65 for ADT+ZQD), the mean ages were 66 for ADT group and 65 for ADT+ZQD (p=0.508). All patients in the two groups had a mean Karnofsky score of 100 (p=0.914). The numbers of patients with a Gleason score of less than 7 were 35 and 32 in ADT and ADT+ZQD groups, respectively (p=0.796). PSA levels were 37.6 and 42.8 ng/mL for ADT and ADT+ZQD groups, respectively (p=0.699). The TNM stages and EORTC QLQ-C30 life quality scores were also comparable between the two groups (Table 1).

**Table 1:** Patient baseline characteristics.

		**ADT (n=68)**	**ADT+ZQD (n=65)**	**P value**
Age (years)		66 (53–75)	65 (50–74)	0.508
Karnofsky score		100 (80-100)	100 (70-100)	0.914
Gleason score	≤7	35 (51.5)	32 (49.2)	0.796
	8-10	33 (48.5)	33 (50.8)	
PSA (ng/ml)		37.6 (73.1)	42.8 (81.1)	0.699
T category	T1 or T2	11	9	0.707
	T3 or T4	57	56	
N category	N0	37	38	0.638
	N1	31	27	
M category	M0	29	29	0.819
	M1	39	36	
EORTC QLQ–C30 (0-100)		61.2 (21.9)	63.2 (19.8)	0.581

Data are median (range), n (%), or mean (SD). ADT, androgen-deprivation therapy. ZQD, Zhoushi Qiling decoction. PSA, prostate-specific antigen.

### ADT and ZQD combinational therapy improved survival of prostate cancer patients

**Figure 2:**
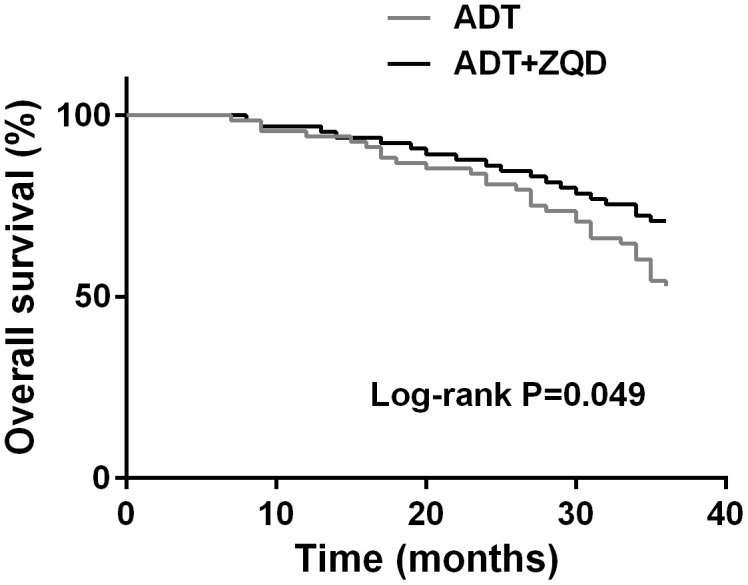
Kaplan-Meier curves estimates the 3-year overall survival of ADT (n=68) and ADT+ZQD (n=65) groups.

At the end of the three-year treatment, the Kaplan-Meier curves were used to estimate the 3-year overall survival of patients in the two groups. As shown in Figure 2, ADT+ZQD treatment resulted in a significantly improved survival compared to ADT treatment alone (p=0.034). The deaths of ADT group and ADT+ZQD group were 43/72 and 19/67, respectively, indicating a strong effect of ADT+ZQD treatment in reducing the cancer-related mortality.

### ZQD treatment upregulates miR-143 expression

**Figure 3:**
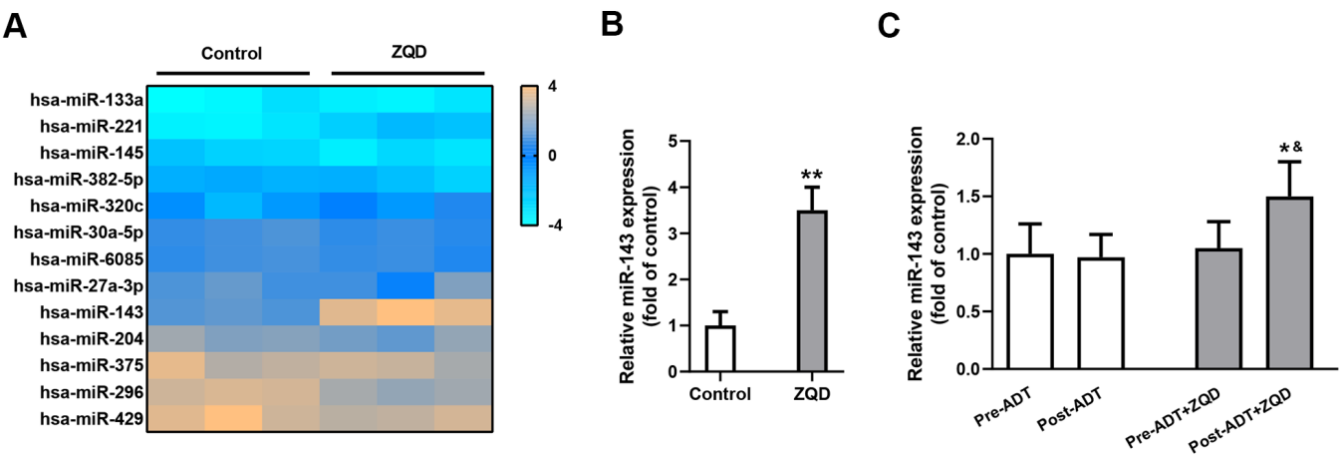
**Zhoushi Qiling decoction modulates the expression of miR-143 in DU145 human prostate cancer cells and patient serum samples.** (**A**) Relative expression levels of miRNAs in DU145 were checked by microarray after 24 hours of treatment with 50 mg/mL ZQD. (**B**) miR-143 expression levels in DU145 cells were analyzed by qRT-PCR. Data were expressed as mean ± SD from three independent experiments with triple replicates per experiment. ** p < 0.01 compared with control group. (**C**) miR-143 expression levels in patient serum samples of ADT (n=68) and ADT+ZQD (n=65) groups were analyzed by qRT-PCR. Data were expressed as mean ± SD. * p < 0.05 compared with post-ADT group. ^&^p < 0.05 compared with pre-ADT+ZQD group.

To clarify the mechanism of action of ZQD, we incubated DU145 cells with 50 mg/mL ZQD and analyzed the expression levels of an array of miRNAs before and after treatment using a microarray assay (Figure 3A). Among the miRNAs investigated, only miR-143 demonstrated prominent upregulation after ZQD treatment. We then proceeded to confirm the upregulation of miR-143 in DU145 cells using qRT-PCR analysis and found that ZQD treatment led to an over three-fold increase in miR-143 expression *in vitro* (p<0.01, Figure 3B). It is worth noting that we did not perform ADT treatment on DU145 cells because DU145 is an androgen-independent cell line which would not respond to ADT therapy [6]. In patient serum samples, miR-143 upregulation was also observed in patient who received ADT+ZQD treatment (p<0.01), while ADT treatment alone did not result in such upregulation (Figure 3C).

### ZQD treatment induces cell apoptosis

**Figure 4:**
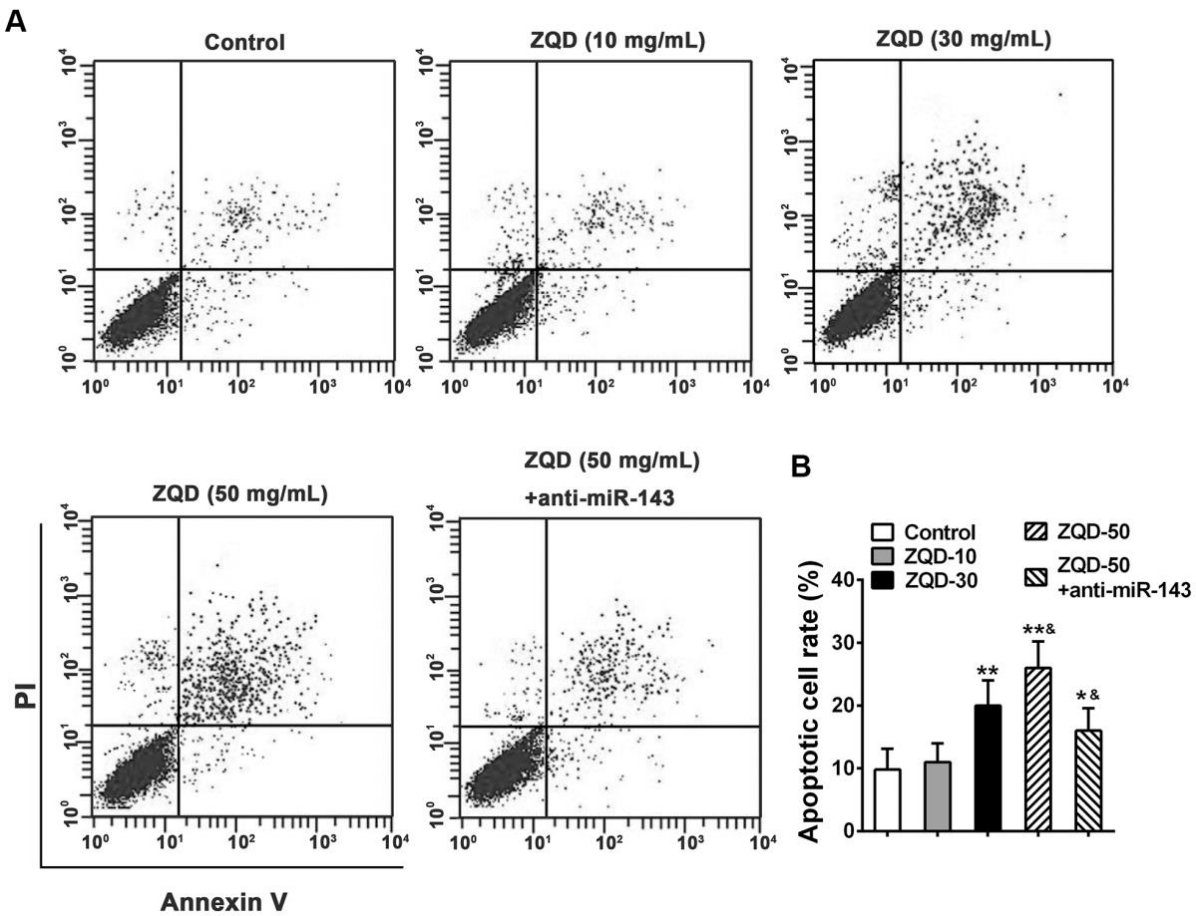
**Zhoushi Qiling decoction induces apoptosis of DU145 human prostate cancer cells after 24 hours of treatment.** (**A**) Apoptotic cells were evaluated by PI and Annexin V staining assay. (**B**) Apoptotic cell rate was expressed as the percentage of total cells. Data were expressed as mean ± SD from three independent experiments with triple replicates per experiment. * p < 0.05, ** p < 0.01 compared with control group. ^&^p < 0.05 compared with ZQD-50 group.

We then tested the efficacy of ZQD in inducing cell apoptosis using a flow cytometry analysis. As shown in Figure 4, we observed that treatment with ZQD of increasing concentrations (10-50 mg/mL) resulted in a dose-dependent increase in cell apoptosis (Figure 4A), and at the dose of 30 and 50 mg/mL, the increase in apoptosis was significant (p<0.01 for 30 mg/mL and p<0.001 for 50 mg/mL) (Figure 4B). Treatment with anti-miR-143 and 50 mg/mL ZQD, in contrast, demonstrated attenuated cell apoptosis rates (p<0.01, compared to treatment with 50 mg/mL ZQD alone). This data suggested that ZQD promoted cell apoptosis but downregulating miR-143 could at least partially attenuate this effect (Figure 4B).

### The pro-apoptotic effects of ZQD are mediated through the interaction between miR-143 and Bcl-2

**Figure 5:**
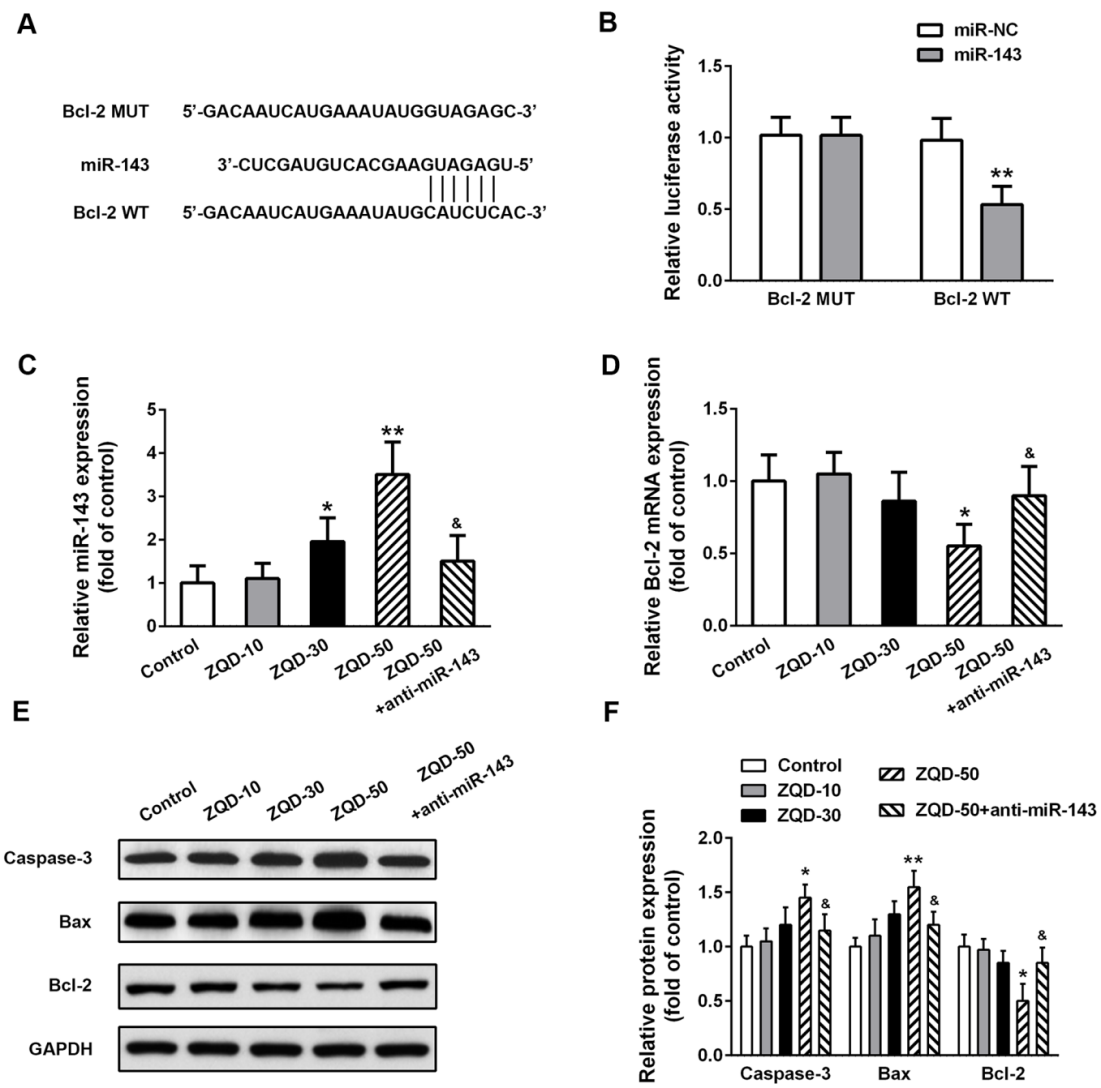
**Zhoushi Qiling decoction effects caspase-3 activity and Bax and Bcl-2 expression levels via miR-143 in DU145 cells.** (**A**) Bcl-2 was predicted as a direct target of miR-143 by the online software Targetscan. (**B**) Luciferase reporter assay was conducted in DU145 to verify the direct binding between miR-143 and Bcl-2. Relative miR-143 (**C**) and Bcl-2 mRNA (**D**) levels were detected by qRT-PCR. Western blot was performed to measure the protein expression of caspase-3, Bax and Bcl-2 (**E**) in different groups and relative protein expression levels (**F**) were quantified. Data were expressed as mean ± SD from three independent experiments with triple replicates per experiment. * p < 0.05, ** p < 0.01 compared with control group. ^&^p < 0.05 compared with ZQD-50 group.

To further elucidate the mechanism of ZQD in inhibiting prostate cancer and unveil the role of miR-143 in mediating the effects of ZQD, we performed bioinformatics analysis using Targetscan, which suggested a direct binding site between miR-143 and the 3’-UTR of Bcl-2 (Figure 5A). Luciferase assay validated the interaction between miR-143 and wild-type Bcl-2, but not mutated Bcl-2 (Figure 5B), suggesting that miR-143 efficiently inhibited Bcl-2 (p<0.01). As ZQD dose-dependently upregulated miR-143, anti-miR-143 decreased miR-143 levels (Figure 5C). As a result, ZQD dose-dependently decreased Bcl-2 expression, which was however abrogated by anti-miR-143 (Figure 5D). Consistently, Western blot analysis suggested that ZQD upregulated Caspase-3 and Bax, while downregulated Bcl-2 (Figure 5E, 5F). These results supported the important role of Bcl-2 and miR-143 interaction in promoting the cytotoxic effects of ZQD.

## DISCUSSION

TCMs have been extensively used in the treatment of patients with HIPC, and the efficacy of TCM stems from the synergistic actions of its complex components [7–9]. But the unclear mechanism of action is a major hurdle in the clinical utility of most TCMs. With the aim of advancing the use of one of the TCMs used in prostate cancer therapy, ZQD, we conducted a randomized controlled trial to corroborate the efficacy of ZQD in HIPC. Due to resistance to ADT therapy, patients with HIPC have a poor survival rate and very few treatment options are available. Our study investigated the effects of ADT+ZQD therapy on HIPC patients and after three years, we found a significantly improved survival rate in patients who received ADT+ZQD therapy compared to those who received ADT therapy alone.

While a few trials have suggested the effects of ZQD in improving prostate cancer patient survival rate, to our best knowledge, this is the first study to investigate the molecular mechanism of ZQD in prostate cancer. Previously, the regulatory role of TCMs against cancer has been mostly limited to inflammation, hematopoiesis, immunity [10], etc., and how miRNAs participate in this regulation is largely unknown. In our study, by examining the expression profile of a number of miRNAs before and after ZQD treatment in DU145 cells, we demonstrated that ZQD was efficient in upregulating miR-143, a putative tumor suppressor [11, 12]. Here we adopted the DU145 cell line, because DU145 is a widely used androgen independent cell line due to the lack of androgen receptors [13]. The upregulation of miR-143 was also observed in patient serum samples after ADT+ZQD treatment. In contrast, patients who received ADT treatment alone did not show miR-143 upregulation, confirming that the upregulation of miR-143 was specifically induced by ZQD. MiR-143 has been reported as a tumor suppressor that targets a large array of cancer-related signaling pathways [14–17], such as KRAS pathway [17], ERK signaling [14], glycolysis [12] and matrix metalloproteinases [18]. Deregulation of miR-143 is associated with tumorigenesis and poor patient survival. However, miR-143 dysregulation has not be associated with the androgen independence of prostate cancer, despite that the interaction between miR-143 and Bcl-2 has been implicated in other cancers [19]. In our study, we showed that Bcl-2, which is associated with the emergence of androgen independence of prostate cancer [20, 21], is a target of miR-143 using bioinformatics analysis, which is further confirmed by luciferase assay. It was found that Bcl-2 exerted an anti-apoptotic effect and Bcl-2 upregulation was linked to poor prognosis in many cancers. Bcl-2 also promotes the transformation of prostate cancer cells from an androgen-dependent to an androgen-independent growth stage. Hence, the upregulation of miR-143 induced by ZQD results in attenuation of Bcl-2 expression, which enhances the therapeutic effects of ADT therapy. Bcl-2 is an important oncotarget in prostate cancer and miRNAs are a class of therapeutics commonly exploited to downregulate Bcl-2. Our data suggested that ZQD therapy was an alternative and clinically applicable approach to modulate the expression of Bcl-2. In addition, we also showed that ZQD itself induced pro-apoptotic effects in DU145 cells as revealed by flow cytometry analysis and upregulation of Caspase-3 and Bax2 protein levels in ZQD treated cells. Together, this evidence indicates that the efficacy of ZQD stems from its direct regulation on prostate cancer cells, i.e., inducing cell apoptosis and sensitizing cells to ADT therapy, corroborating the potency of ZQD as an effective TCM in HIPC.

The study is nevertheless limited by its relatively small sample size (only 133 patients were included for analysis). Also, the clinical trial was not blinded, and placebo control could be useful to corroborate the efficacy of ZQD as a combinational therapy to ADT. Here we did not investigate whether ADT+ZQD therapy led to improvement on tumor stage, physical conditions and quality of life of patients, which are also important parameters to evaluate the benefit of ADT+ZQD therapy. In addition, as studies on the optimal dose of ZQD are rare, we only tested the dose of 100 mL of ZQD extract taken twice daily to treat patients. Further optimization of the ZQD dose is also needed to maximize the patient survival. Together, further studies are warranted to comprehensively evaluate the effects of ZQD on these parameters to guide the use of ZQD therapy in prostate cancer patients.

## CONCLUSIONS

Here we have conducted a randomized controlled clinical trial to corroborate the efficacy of ZQD as a combinational therapy to ADT in improving survival of prostate cancer patients. ZQD dose-dependently upregulates miR-143 and induces cell apoptosis. Since Bcl-2 is a target of miR-143, ZQD therapy downregulates Bcl-2 and upregulates Caspase-3 and Bax2 to suppress prostate cancer progression.

## MATERIALS AND METHODS

### Patient enrollment

The clinical trial was approved by the ethics committee of LONGHUA Hospital Shanghai University of Traditional Chinese Medicine. All participants provided informed consent. Inclusion criteria were: 1) patients diagnosed with advanced prostate cancer; 2) complete medical records; 3) aged 50-75; 4) prognosis > 3 months; 5) no radiation therapy, chemotherapy or other treatment within 4 weeks before this trial.

The clinical trial number of this study is ChiCTR2000039072.

### Treatment procedures

Patients were randomly assigned into ADT and ADT + ZQD groups. ZQD treatment: the ZQD was formulated as the following: 15 g of Astragalus mongholicus Bunge, 30 g of Rabdosia rubescens Hara, 15 g of Radix Rehmanniae, 12 g of radix codonopsis, 9 g of rhizoma curcumae longae, 15 g of Solanumseptemlobum Bunge, 15 g of Rhizoma Curcumae Longae, 9 g of Herba Leonuri, and 9 g of Radix Glycyrrhizae Preparata. ZQD was boiled and the liquid extract of 200 mL was packed in two vacuumed containers (100 mL in each container). Then, ZQD was orally administered twice daily in the morning and at night with each dose of 100 mL. Treatment lasted for 3 months. At the same time, ADT (leuprorelin 3.75 mg) was injected subcutaneously in the anterior abdominal wall once a month for three years. The follow-up period was 3 years.

### Cell culture and treatment

The DU145 prostate cancer cell line was acquired from American Type Culture Collection (Rockville, MD, USA) and cultured in RPMI medium supplemented with 10% fetal bovine serum. DU145 was treated with ZQD (0, 10, 30 50 mg/mL) for 24 h. In another group, DU145 cells transfected with anti-miR-143 (synthesized by GenePharm Inc., China) were treated with 50 mg/mL ZQD for 24 h as control.

### Quantitative real-time PCR

qRT-PCR was performed to analyze the expression of miR-143 and Bcl-2. Trizol Agent (Invitrogen, USA) was used to extract the total RNA from DU145 cells and human serum. RT-PCR was performed using cDNA synthesis kit (ThermoFisher, USA) and SYBR green reagents (ThermoFisher, USA). Primers for miR-143, Bcl-2 and GAPDH were synthesized by GenePharm (China). The expression levels were normalized to GAPDH.

### Apoptosis analysis

Cell apoptosis was assessed by Annexin V-FITC-PI staining flow cytometry in a flow cytometer.

### Western blot

Cells were lysed using RIPA buffer and BCA assay was used to quantify protein concentration. Protein loaded to 12% SDS gel was used for electrophoresis, followed by transferring to PVDF membrane. BSA (1%) was used to block the membrane. Primary antibodies against Caspase-3, Bcl-2, Bax and GAPDH, and HRP-conjugated secondary antibody were acquired from Abcam (USA). GAPDH was used as a loading control.

### Statistical analysis

Data were presented with mean ± SD. The Student’s t-test, or one-way ANOVA analysis followed by a Tukey post hoc test was performed to compare difference between groups.
